# Remora Namib Beetle Optimization Enabled Deep Learning for Severity of COVID-19 Lung Infection Identification and Classification Using CT Images

**DOI:** 10.3390/s23115316

**Published:** 2023-06-03

**Authors:** Amgothu Shanthi, Srinivas Koppu

**Affiliations:** School of Information Technology and Engineering, Vellore Institute of Technology, Vellore 632014, India; amgothu.shanthi2020@vitstudent.ac.in

**Keywords:** deep quantum neural network (DQNN), deep neuro fuzzy network (DNFN), pyramid scene parsing network (PSP-Net), Remora optimization algorithm (ROA), Namib beetle optimization (NBO)

## Abstract

Coronavirus disease 2019 (COVID-19) has seen a crucial outburst for both females and males worldwide. Automatic lung infection detection from medical imaging modalities provides high potential for increasing the treatment for patients to tackle COVID-19 disease. COVID-19 detection from lung CT images is a rapid way of diagnosing patients. However, identifying the occurrence of infectious tissues and segmenting this from CT images implies several challenges. Therefore, efficient techniques termed as Remora Namib Beetle Optimization_ Deep Quantum Neural Network (RNBO_DQNN) and RNBO_Deep Neuro Fuzzy Network (RNBO_DNFN) are introduced for the identification as well as classification of COVID-19 lung infection. Here, the pre-processing of lung CT images is performed utilizing an adaptive Wiener filter, whereas lung lobe segmentation is performed employing the Pyramid Scene Parsing Network (PSP-Net). Afterwards, feature extraction is carried out wherein features are extracted for the classification phase. In the first level of classification, DQNN is utilized, tuned by RNBO. Furthermore, RNBO is designed by merging the Remora Optimization Algorithm (ROA) and Namib Beetle Optimization (NBO). If a classified output is COVID-19, then the second-level classification is executed using DNFN for further classification. Additionally, DNFN is also trained by employing the newly proposed RNBO. Furthermore, the devised RNBO_DNFN achieved maximum testing accuracy, with TNR and TPR obtaining values of 89.4%, 89.5% and 87.5%.

## 1. Introduction

Medical imaging techniques have become an important progression in recent years and have generally been utilized for diagnosing and quantifying several diseases. The most generally utilized diagnosing kit for COVID-19 infections is by means of RT-PCR assays of nasopharyngeal swabs [1]. The substitute solution to reverse-transcription polymerase chain reaction (RT-PCR) for screening of COVID-19 is medical imaging methods, namely computed tomography (CT) or X-ray [2,3]. The CT and a chest X-ray (CXR) images are highly suited to identify lung infections due to COVID-19. While comparing them to swab analysis, CT as well as CXR identify the spatial position of suspicious disease and the extent of the damages. A hallmark illness of CXR is the bi-lateral distribution of peripheral unclear lung opacities, including the consolidation of air space [4]. Mainly, the screening of chest CT has emerged as a regular tool for diagnosing pneumonia. Hence, chest CT images have been highly suggested for the diagnosis of COVID-19. Additionally, CT imaging plays a significant role in the monitoring of diseases and quantification analysis of COVID-19. The infected regions of COVID-19 are noticeable on CT images by the ground glass opacity (GGO) in an earlier stage of infection and by means of pulmonary consolidation in a later stage of infection [2,3]. Moreover, CT imaging receives increasing attention from the research community [5], wherein efforts have been undertaken for the investigation of COVID-19 pathology alteration from the viewpoint of radiology [6]. The benefit of imaging is that it achieves better sensitivity, rapid turn-around time and is able to view the extent of the lung infection [4].

In December 2019, a few hospitals in the city of Wuhan, Hubei region, found numerous cases of inexplicable pneumonia in patients having had contact with a sea-food market in Southern China. It had been affirmed as acute respiratory infection, which is caused by means of the 2019 new coronavirus (2019-nCoV) [7]. A pneumonia thus caused by 2019-nCoV was termed as COVID-19 by the World Health Organization (WHO). COVID-19 became a global pandemic with numerous positive cases as well as thousands of corresponding deaths [8]. The count of infections and corresponding deaths is still increasing rapidly everyday across the world [9]. The ordinary COVID-19 symptoms are diarrhoea, painful throat, fever, headaches, coughing and shortness of breath. Taste vanishing, aches, nasal blocking, tiredness and loss of smell are also some of the symptoms observed in the affected patients. This virus was also named as SARS-CoV-2 by the International Committee on Taxonomy of Viruses (ICTV) [10,11]. The rapid spreading of this disease can be slowed down or stopped by means of quarantine and suitable treatments. In accordance to the clinical management of WHO, SARS-CoV-2 RT-PCR assessment of the upper respiratory tract specimen is highly suggested for suspicious cases. On the other hand, ribonucleic acid (RNA) examination can be utilized for the identification of infected patients with COVID-19, but the degree of infection for the patients remains undetected. Hence, it is complicated to perform targeted treatment [9]. RT-PCR is capable of identifying viral RNA in the specimens acquired by bronchoalveolar lavage, tracheal aspirate, nasopharyngeal swab or oropharyngeal swab [2].

The utilization of deep learning is steadily raising and is employed in several applications such as automated driving, face recognition, machine learning and image processing in medical fields. It is an efficient technique, which utilizes deep learning for the classification of COVID-19 and lesion segmentation. Many important techniques are developed for COVID-19 identification and detection [12,13]. For the patients affected with COVID-19, various infection degrees greatly influence the treatment. Accurately segmenting diverse kinds of lung-infected regions could help the doctors in identifying a particular treatment. However, accurate COVID-19 segmentation is very complicated due to several reasons [9]. An image assessment for COVID-19 provides a better solution for earlier identification, owing to complications in laboratory evaluation and the significance of earlier detection for saving lives. Similarly, several techniques have been devised for COVID-19 detection and control utilizing CT or X-ray lung images. Moreover, the utilization of artificial intelligence (AI) makes the performance and precision of these outcomes convincing. By utilizing AI tools as well as computer vision approaches, the detection accuracy is accurate with maximum precision [14]. Many deep convolutional neural networks such as COPLE-Net [15] have been developed and proved their capabilities in offering the best performance on infection segmentation of COVID-19 from lung CT images [16]. For CT imaging, the location-attention oriented method was utilized [17] for calculating the infection possibility of COVID-19. The weakly supervised deep learning-enabled software method is developed [18] utilizing 3D CT images for the detection of COVID-19. Therefore, deep learning approaches play a significant role in the identification and classification of COVID-19 lung infection.

The preeminent intention of this work is to design proficient techniques for COVID-19 lung infection identification and classification using CT images, which are named as RNBO_DQNN and RNBO_DNFN. Firstly, lung CT images are acquired from a dataset [19], which is fed to pre-processing wherein an adaptive Wiener filter is utilized for noise removal and a region of interest (ROI) is extracted for further processing. Thereafter, lung lobe segmentation is performed based on PSP-Net [20]. Afterwards, feature extraction is carried out in which a local ternary patten (LTP) [21], a local Gabor XOR pattern (LGXP), a grey-level co-occurrence matrix (GLCM) feature [22] and statistical features, such as mean, variance, standard deviation, kurtosis and skewness are extracted. Then, the first level of classification is conducted employing DQNN [23], wherein extracted features are classified as COVID-19 or non-COVID-19. Furthermore, DQNN is trained by the proposed RNBO, which is newly presented by merging ROA [24] and NBO [25]. If the classified output is COVID-19, then the second level of classification is carried out using DNFN [26], wherein the affected areas due to COVID-19 are further classified as low, moderate and high infection affected areas in the lungs [27,28]. Additionally, DNFN is also trained employing the newly introduced RNBO.

The prime contribution of this work is revealed below.

Devised RNBO_DQNN for the identification of COVID-19 lung infection: In this work, the proficient approach called RNBO_DQNN is presented for the identification of COVID-19 lung infection. In a first level of classification, DQNN is used for identifying the disease as COVID-19 or non-COVID-19. Moreover, DQNN is tuned by RNBO, which is an combination of ROA and NBO.

Proposed RNBO_DNFN for classification of COVID-19 lung infection: Here, an efficacious method termed as RNBO_DNFN is introduced for the classification of COVID-19 lung infection. If the output identified is COVID-19 in the first level, then the second level of classification is carried out by DNFN to further categorize into low, moderate or high infection affected areas in the lungs. Moreover, DNFN is tuned by RNBO, which is an combination of ROA and NBO.

The remaining sections’ arrangement is as follows: Section 2 describes the literature review of traditional approaches and Section 3 elucidates the steps for the proposed technique, while Section 4 discusses the results for two levels of classification and Section 5 reveals the conclusion of this work.

## 2. Motivation

As COVID-19 spreads globally, the detection of infected lung regions from CT images provides high potential for increasing classical healthcare planning to tackle COVID-19. In the existing techniques, the infected lung regions are frequently unclear and thus it is very complicated for doctors as well as machines to differentiate normal images from infected images. This motivated us to devise an approach for the identification of lung infected areas in CT images of COVID-19-affected patients. This segment presents the existing works related to COVID-19 lung infection identification as well as classification with limitations.

### 2.1. Literature Survey

A literature study conducted by several existing approaches for COVID-19 lung infection and classification are interpreted as follows. Deng-Ping Fan et al. [29] introduced a COVID-19 Lung Infection Segmentation Deep Network (Inf-Net) for the automatic identification of infected areas in CT images. This method improved the learning capability and achieved maximum performance, but it did not conduct two-phase planning to attain multi-class infection labelling, which may result in sub-optimum learning performance. Lal Hussain et al. [4] developed a machine learning classification of texture features for the identification of COVID-19 lung infection, which greatly increased the positive prediction values but failed to include further lung disorders. Dominik Muller et al. [2] utilized 3D U-Net for the automatic segmentation of infected areas due to COVID-19 in CT images and enhanced the medical image assessment with less data, even though it did not investigate evaluation enhancement probabilities. Omar Elharrouss et al. [14] presented a multi-task deep-learning-based technique which attained a higher degree of performance with a lack of data as well as labelled images, though it failed to utilize more data for tuning and several labelled data for the multi-class segmentation.

Ramin Ranjbarzadeh et al. [10] developed a technique for the segmentation of lung infection from COVID-19 on the basis of a cascade convolutional neural network (CNN). This technique efficiently resolved the over-fitting problems but failed to decrease training time. Jiannan Liu et al. [6] presented a transfer learning technique for the segmentation of COVID-19 lung infection, which highly increased the segmentation accuracy as well as the generalization capability, though it did not consider independent and scattered pulmonary nodules for segmentation. Bingbing Zheng et al. [9] introduced a multi-scale discriminative network (MSD-Net) for the segmentation of multiple classes of COVID-19 lung infection on CT images. In this technique, the segmentation performance was highly increased, but owing to an inadequate sample count, mild symptoms were vague and hard to differentiate. Jianning Chi et al. [16] developed a multi-input directional UNet (MID-UNet) for COVID-19 lung infection segmentation from the CT images. This technique decreased the processing time, but it still did not include a few multi-class labelling approaches for differentiating diverse kinds of infected regions.

### 2.2. Challenges

In addition to the advantages of the existing approaches, some disadvantages remain. The few disadvantages experienced by the reviewed techniques are as follows.

➢ The Inf-Net model in [29] focused on COVID-19 lung infection segmentation for affected patients. This approach failed in terms of clinical management as it frequently needed to classify the COVID-19 patients and then segment the infected areas for additional treatment.
➢ U-Net utilized in [2] for the segmentation of COVID-19 affected regions failed to conduct semantic segmentation in clinical management for estimating the clinical robustness and performance.
➢ The technique developed in [10] to segment lung infection from COVID-19 did not differentiate pulmonary nodules inside a normal lung near a lesion border from infected tissue.
➢ The transfer learning approach in [6] was presented for the segmentation of COVID-19 lung infection, but it still did not decrease the computational complications.
➢ The coronavirus pandemic affected many people across the world and created great challenges for international health management. Hence, continual screening, earlier diagnosis and timely actions are significant for controlling the spread as well as reducing mortality.

## 3. Proposed RNBO_DQNN and RNBO_DNFN for Identification and Classification of COVID-19 Lung Infection

**Figure 1:**
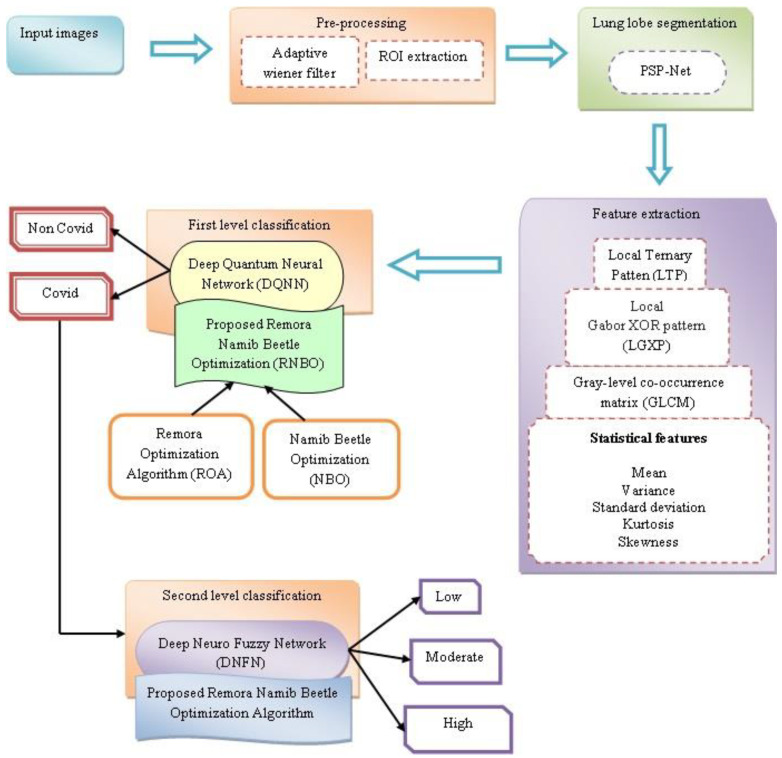
Pictorial representation of proposed techniques for identification and classification of COVID-19 lung infection.

An exact segmentation of diverse kinds of lung infection regions could help the doctors to prescribe a particular treatment. Moreover, the exact segmentation of COVID-19 is a complicated feat due to several reasons. Therefore, the approaches, namely RNBO_DQNN and RNBO_DNFN, are effectively introduced for the identification and classification of COVID-19 lung infection. At first, lung CT images are acquired and thereafter pre-processing is performed, wherein an adaptive Wiener filter is utilized and an ROI is extracted for further processing. After that, lung lobe segmentation is performed employing PSP-Net. Thereafter, feature extraction is conducted for the extraction of features to perform classification phases. Then, DQNN is utilized to identify COVID-19 or non-COVID-19 from the extracted features. If the detected output is COVID-19, the infected regions are categorized as low, moderate and high infection in the lungs by DNFN. Moreover, DQNN and DNFN are trained by the proposed RNBO, which is newly introduced by combining ROA and NBO. Figure 1 demonstrates the graphic representation of devised techniques for the identification and classification of COVID-19 lung infection.

### 3.1. Acquisition of Input Image

Considering CT images for the identification and classification of COVID-19 lung infection, which are attained from the specific database mentioned in [19], can be modelled by

(1) $$C=\left\{M_{1},M_{2},\ldots M_{l}\ldots M_{i}\right\}$$

Here, the total number of training images contained in database C is represented by i, where $M_{l}$ signifies lth input image.

### 3.2. Image Pre-Processing Using Adaptive Wiener Filtering and ROI Extraction

Pre-processing is a vital step in the image-processing approaches, where an image is denoised without affecting its quality utilizing filters. Here, adaptive Wiener filtering is used for the elimination of noises and artifacts from input images, and then ROI extraction is carried out.

#### 3.2.1. Adaptive Wiener Filtering

An adaptive Wiener filtering [30] regulates the output pf filter in accordance with the local variance of an image. The major objective is to reduce the mean square error among restored images as well as actual images. The filtering outcome of this technique is better than other filtering methods as well as being highly helpful for preserving edges and higher-frequency areas of an image. Moreover, the artifacts present in the images are removed using the adaptive Wiener filtering.

Let us consider that the filtering of the images corrupted by means of signal intendent noise and can be formulated by

(2) $$\beta \left(y,z\right)=\eta \left(y,z\right)+\alpha \left(y,z\right)$$

Here, noisy measurement is denoted by $\beta \left(y,z\right)$, noise-free image is represented by $\eta \left(y,z\right)$ and additive noise is symbolized by $\alpha \left(y,z\right)$. An intention is to eliminate noise $\beta \left(y,z\right)$. For the pixel in an image, variance and mean of pixel in diverse window dimensions such as ${\left(3+2y\right)}^{2},\;y=0,1,2,3$ are compared and thereafter the window utilized by the minimal average value is considered as the last processing window. The template for the filter can be chosen adaptively in accordance with diverse areas. The smaller window filter is utilized in the brief portion and the larger window filter is utilized in the smooth region, which can enhance the efficacy and retain texture portions as well as edges. The formula utilized for processing the pixels to obtain output outcomes is illustrated by

(3) $$\nu \;\left(y,z\right)=\chi +\left(1-x+\Delta \right)\times \left(g\left(y,z\right)-\chi \right)$$

(4) $$x=\frac{\sigma_{\gamma }}{\sigma_{\rho }+1}$$

(5) $$\Delta =\frac{\sigma_{\rho }}{\sigma_{\gamma }+\sigma_{\max}+1}$$

Here, the original pixel is given by $g\left(y,z\right)$ and $\nu \left(y,z\right)$ signifies an output pixel, whereas all the pixels’ mean variance in the chosen window is symbolized by $\sigma_{\gamma }$, $\sigma_{\rho }$ reveals the present pixel’s variance and the maximal variance of every pixel in an image is denoted by $\sigma_{\max}$.

#### 3.2.2. ROI Extraction

An input for ROI extraction is a filtered image signified by $\nu \left(y,z\right)$. ROI extraction is the crucial pre-processing step in case of image assessment and classification. The appropriate extraction of the ROI could enhance the odds of the medical diagnosis technique correctly by detecting significant regions in an image with an occurrence of COVID-19, as it rejects non-associated information. Therefore, a pre-processed image is symbolized by $R_{l}$.

### 3.3. Lung Lobe Segmentation Using PSP-Net

The lobes are the most independent anatomical compartment of the lungs, which play a significant role in the diagnosis and treatment of lung diseases. The exact location of the lobe separating fissures in CT images frequently indicates a non-trivial chore, even for specialists. Here, lung lobe segmentation is conducted employing PSP-Net and a pre-processed image $R_{l}$ is provided as input. PSP-Net offers a better trade-off among the localization object’s precision and accuracy of segmentation. It offers effective results in the segmentation.

#### Architecture of PSP-Net

PSP-Net [20] is a semantic segmentation system, which utilizes a pyramid pooling module that uses global context information by diverse area-enabled context aggregation.

A pyramid pooling module combines features by means of four several pyramid scales. The coarsest level is a global pooling for generating individual bin output. The pyramid level divides a feature map into diverse sub-areas and presents a pooled depiction for diverse positions. An output of diverse phases in the pyramid pooling module comprises a feature map of various sizes. For maintaining a weight of global features, a 1×1 convolution layer is utilized after each of the pyramid levels for reducing the size of the context depiction to 1/Ζ of real one if a level dimension of the pyramid is Ζ. Thereafter, lower-size feature maps are directly up-sampled in order to obtain similar dimension features as a real feature map through bilinear interpolation. Lastly, a diverse level of features is concatenated as the last pyramid pooling global feature.

**Figure 2:**
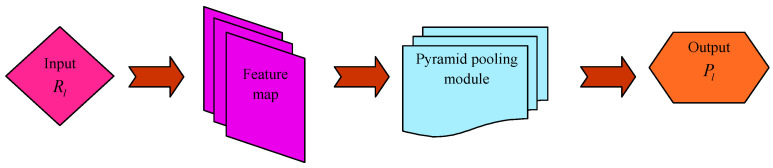
Architecture of PSP-Net.

For a given input image, the pre-trained ResNet model along with a dilated network system are utilized for extracting the feature map. On the top of the map, a pyramid pooling module is utilized for gathering contextual information. Utilizing a four-level pyramid, the pooling kernel covers an entire as well as few half portions of an image that are combined as global prior. Thereafter, the prior is concatenated with real feature map in the last portion. This is then followed by a convolutional layer for generating the last prediction map. Thus, PSP-Net offers an efficient global context for pixel-level scene parsing. A pyramid pooling module could be able to collect a level of information highly representative of the global pooling. The segmented output acquired from PSP-Net is denoted by $P_{l}$. Figure 2 represents the architecture of PSP-Net.

### 3.4. Feature Extraction

Feature extraction elucidates the related shape information consisting of a pattern and hence, the task of categorizing a pattern is made easier by the formal process. In image processing, feature extraction is a specific way of reducing dimensionality. The segmented output $P_{l}$ is given as the input for the extraction of features, such as LTP, LGXP, GLCM features and statistical features, such as standard deviation, mean, kurtosis, variance and skewness.

#### 3.4.1. LTP

LTP consists of three-valued codes such as $\left(-1,0,1\right)$, where grey levels in the width zone from −w to +w about $q_{b}$ are quantized to zero, whereas below and above are quantized to −1 and 1, respectively. The function $t\left(\psi \right)$ is substituted by a three-valued function, which is illustrated by

(6) $$E_{1}=t\left(v,q_{b},w\right)=\left\{\begin{array}{l} 1,\;v\geq q_{b}+w\\ 0,\;\left|v-q_{b}<w\right.\\ -1,\;v\leq q_{b}-w \end{array}\right.$$

Here, w represents the user-defined threshold. A feature extraction utilizing LTP is more robust and resistant to noise in nearer uniformity areas.

#### 3.4.2. LGXP

The local Gabor XOR patterns (LGXPs) are proposed by encoding the Gabor phase using the local XOR pattern (LXP) operator. The phases of LGXPs [31] are initially quantized into a diverse range, and thereafter an LXP operator is utilized to quantize the phases of the central pixel and of each of their neighbours. Lastly, the resultant binary labels are mutually concatenated as a local pattern of a central pixel.

Normally, an LGXP pattern [29] in decimal and binary form is modelled by

(7) $$E_{2}=L_{\lambda ,\omega }\left(\kappa_{\upsilon }\right)={\left[L_{\lambda ,\omega }^{S},L_{\lambda ,\omega }^{S-1},\ldots ,L_{\lambda ,\omega }^{1}\right]}_{B}={\left[\sum_{j=1}^{S}2^{j-1}L_{\lambda ,\omega }^{j}\right]}_{D}$$

Here, B and D denote binary and decimal, respectively, the central pixel location in the Gabor stage map having a scale ω and orientation λ is signified as $\kappa_{\upsilon }$, whereas the dimension of the neighbourhood is indicated as S and $L_{\lambda ,\omega }^{j}\;\;\left(j=1,2,\ldots ,S\right)$ symbolizes a pattern computed among $\kappa_{\upsilon }$ and the neighbour $\kappa_{j}$, which is formulated by

(8) $$L_{\lambda ,\omega }^{j}=\tau \left(\Phi_{\lambda ,\omega }\left(\kappa_{\upsilon }\right)\right)⊗\tau \left(\Phi_{\lambda ,\omega }\left(\kappa_{j}\right)\right),\;\;\;\;\;\;\;\;\;\;\;\;\;\;\;\;\;\;j=1,2,\ldots ,S$$

Here, phase is denoted by $\Phi_{\lambda ,\omega }\left(•\right)$ and the LXP operator is signified by ⊗, which is based on the XOR operator. $\tau \left(•\right)$ symbolizes the quantization operator which evaluates quantized code of phases in accordance with the count of phase ranges, which can be modelled by

(9) $$\upsilon ⊗u=\left\{\begin{array}{l} 0,\;\;\;\;\;\;\;if\;\upsilon =u\\ 1,\;\;\;\;\;\;\;\;else \end{array}\right.$$

(10) $$\tau \left(\Phi_{\lambda ,\omega }\left(•\right)\right)=j;\;\;\;if\;\frac{360*j}{l}\leq \Phi_{\lambda ,\omega }\left(•\right)<\frac{360*\left(j+1\right)}{l},\;\;\;\;\;\;j=0,1,\ldots .,l-1$$

Here, l indicates the count of phase ranges. Therefore, feature images are obtained from LTP and LGXP features extraction, which is given by

(11) $$O_{l}=\left\{E_{1},E_{2}\right\}$$

#### 3.4.3. GLCM Features

Thereafter, the feature images obtained from LTP and LGXP features extraction signified by $O_{l}$ are applied over GLCM features and statistical features in order to obtain a feature vector.

GLCM is a statistical technique of investigating textures, which considers the spatial association of pixels. GLCM operates by characterizing image texture by computing the pair of a pixel having particular values and the specific spatial association happening frequently in image, developing GLCM and thereafter extracting statistical calibrations from this matrix. The grey co-matrix operation developed GLCM by computing the frequent occurrence of a pixel with an intensity value ε happens in a particular spatial association to a pixel having a value ϑ. The spatial association is defined as a pixel of interest as well as next to the right of the pixel. Each of the components ε,ϑ in the resulting GLCM are a sum of time counts, with a pixel having a value ε occurring in particular spatial association to the pixel having a value ϑ in an input image.

The GLCM matrix comprises a count of columns and rows equal to count of grey levels, A, in an image. A matrix component $Y\;\left(\varepsilon ,\vartheta \;\left|\;\Delta \delta ,\Delta 𝜕\right.\right)$ is a relative frequency, which is divided by means of pixel distance $\left(\Delta \delta ,\Delta 𝜕\right)$. The matrix component is also indicated by $Y\;\left(\varepsilon ,\vartheta \;\left|\;Z,\theta \right.\right)$, which consists of second-order possibility value for alterations among grey levels ε and ϑ at the distance Z with a specific angle θ. Several features are extracted from GLCM, with A being the count of grey levels utilized and μ representing the mean value of Y. $\mu_{\delta },\mu_{𝜕},\sigma_{\delta }$ and $\sigma_{𝜕}$ are means as well as standard deviations of $Y_{\delta }$ and $Y_{𝜕}$. $Y_{\delta }\left(\varepsilon \right)$ signifies εth entry attained by adding the rows of $Y\left(\varepsilon ,\vartheta \right)$.

(12) $$Y_{\delta }\left(\varepsilon \right)=\sum_{\vartheta =0}^{A-1}Y\left(\varepsilon ,\vartheta \right)\;\mathrm{and}\;Y_{𝜕}\left(\vartheta \right)=\sum_{\varepsilon =0}^{A-1}Y\left(\varepsilon ,\vartheta \right)$$

(13) $$\mu_{\delta }=\sum_{\varepsilon =0}^{A-1}\varepsilon Y_{\delta }\left(\varepsilon \right)\;\mathrm{and}\;\mu_{𝜕}=\sum_{\vartheta =0}^{A-1}\vartheta Y_{𝜕}\left(\vartheta \right)$$

(14) $$E_{3}=\sigma_{\delta }^{2}=\sum_{\varepsilon =0}^{A-1}{\left(Y_{\delta }\left(\varepsilon \right)-\mu_{\delta }\left(\varepsilon \right)\right)}^{2}\;\mathrm{and}\;\sigma_{𝜕}^{2}=\sum_{\vartheta =0}^{A-1}{\left(Y_{𝜕}\left(\vartheta \right)-\mu_{𝜕}\left(\vartheta \right)\right)}^{2}$$

#### 3.4.4. Statistical Features

Statistical features, that is to say, mean, kurtosis, standard deviation, variance and skewness, are extracted and elucidated in the subsections below.

(i) Mean

Mean is specified as the overall image values divided by the overall count of pixel values. It can be indicated by $E_{4}$ and calculated as

(15) $$E_{4}=\frac{1}{i}\sum_{l=1}^{i}O_{l}$$

Here, i represents the total count of images and the extracted output is symbolized by $O_{l}$.

(ii) Variance

The square of standard deviations in an output or input image values, where variance can be computed as shown in the below equation.

(16) $$E_{5}=V_{a}{}^{2}=\frac{1}{i}\sum_{l=1}^{i}\left(O_{l}-E_{4}\right)$$

where $E_{4}$ specifies the mean.

(iii) Standard deviation

Standard deviation can be specified to a square root of the variance, which can be expressed as follows:

(17) $$E_{6}=\sqrt{\frac{1}{i}\sum_{l=1}^{i}\left(O_{l}-E_{4}\right)}$$

where the standard deviation is represented by $E_{7}$.

(iv) Kurtosis

The statistical computation used to describe experimented image distribution around a mean is known as kurtosis. It is formulated by

(18) $$E_{7}=\frac{\sum {\left(E_{4}-\bar{E_{4}}\right)}^{4}}{iE_{7}}$$

Here, $E_{4}$ represents mean and $E_{7}$ indicates standard deviation.

(v) Skewness

Skewness defines a measure of images distorted from the actual images and can be expressed by the following equation.

(19) $$E_{8}=\frac{\sum_{l}^{i}\;\left(\rho_{i}-\bar{E_{4}}\right)}{\left(i-1\right)*E_{6}}$$

Here, i denotes the count of images and $\rho_{i}$ indicates a randomly chosen image.

Therefore, as an output from feature extraction, a feature vector is formed which can be represented by

(20) $$T_{l}=\left\{E_{3},E_{4},E_{5},E_{6},E_{7},E_{8}\right\}$$

### 3.5. Detection of COVID-19 Using RNBO_DQNN (First Level of Classification)

Presently, COVID-19 detection is the major challenge across the globe owing to the rapid spread of the disease. The detection of COVID-19 markers at the lower portions of the lungs can be achieved with high accuracy when CT images are used. Here, the detection of COVID-19 is performed by DQNN which is tuned by employing the devised RNBO. However, RNBO is the combination of ROA and NBO. An input given for the first level of classification is specified by $T_{l}$.

#### 3.5.1. Architecture of DQNN

QNN [23] is the quantum circuit of quantum perceptrons arranged into X hidden layers of qubit depending on the initial state $\phi^{I}$ of input qubits and developing mixed state $\phi^{O}$ generally for output qubits according to

(21) $$\phi^{O}=tr_{I,H}\;{\left(K\left(\phi^{I}⊗{|0...0\rangle }_{H,O}\langle 0...0|\right)\;K^{*}\right)}_{}$$

Here, $K=K^{O}K^{X}K^{X-1}\ldots K^{1}$ is a QNN quantum circuit. $K^{\varpi }$ are layer unitaries contained in quantum perceptron products that depend on qubits in the layers ϖ−1 and ϖ. As perceptrons are arbitrary unitary operators, there is no exchange and therefore the functioning order is important.

Universal quantum calculation directly depends on the quantum circuit configuration of QNN. It is observed that a QNN consisting of quantum perceptrons, which depend on four-level quadits that commute within each layer, is still able to execute universal quantum calculation. Generally, the common form of quantum perceptrons allows for the execution of a quantum channel on input quadits.

The crucial QNN property is an output of the network which can be modelled by composing a sequence of complete positive layers to layer transition maps $\xi^{\varpi }$.

(22) $$\phi^{O}=\xi^{O}\;\left(\xi^{X}\left(\ldots \xi^{2}\left(\xi^{1}\left(\varpi^{I}\right)\right)\ldots \right)\right)$$

(23) $$\xi^{\varpi }\left(W^{\varpi -1}\right)\equiv tr_{\varpi -1}\left(\Pi_{o-s_{\varpi }}^{1}K_{o}^{\varpi }\left(W^{\varpi -1}\right)⊗{|0...0\rangle }_{\varpi }\langle 0...0|\right)\;\;\;\Pi_{0=s_{\varpi }}^{1}K_{o}^{\varpi }$$

Here, $K_{o}^{\varpi }$ denotes the oth perceptron dispensing on the layers ϖ−1 and ϖ. This characteristic of QNN output signifies a vital structural feature and information propagation from an input to an output; hence, it executes a quantum feed forward neural network. The crucial outcome is the basis of the quantum analogue back-propagation approach.

Alternatively, a QNN with a recent plan of quantum perceptrons as one controlled unitary can be defined as

(24) $$K=\sum_{ƛ}|ƛ\rangle \langle ƛ|⊗K\left(ƛ\right)$$

where $|ƛ\rangle$ is the basis for an input space and $K\left(ƛ\right)$ are parameter unitaries. Substituting this statement into Equation (22) specifies that an output state is an outcome of calculation and preparation. It can be given as

(25) $$\sum_{ƛ}\langle ƛ|\phi^{I}|ƛ\rangle K\left(ƛ\right)|0\rangle \langle 0|K{\left(ƛ\right)}^{*}$$

**Figure 3:**
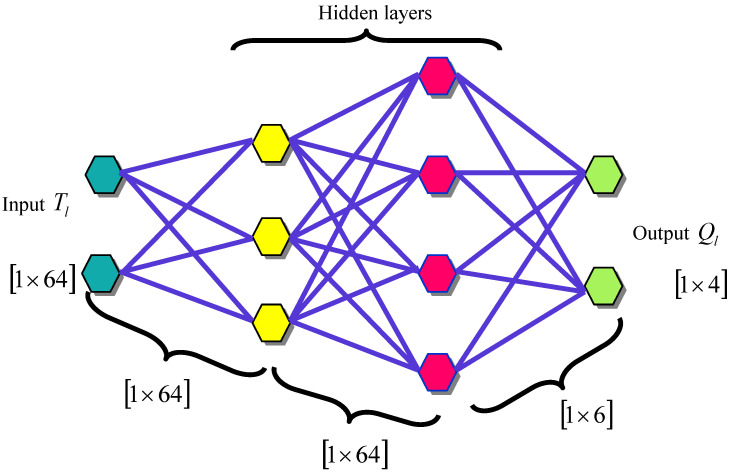
The architecture of DQNN.

These channels do not have a non-zero quantum channels ability and cannot execute general quantum calculation. The DQNN output is signified by $Q_{l}$. The architecture of the DQNN is shown in Figure 3.

#### 3.5.2. Training of DQNN Utilizing Proposed RNBO

ROA is stimulated mainly owing to the parasitic characteristic of the Re mora, and it also offers a highly promising viewpoint and a stronger competitive capability. NBO is based upon the characteristics of Namib beetles from Saharan Africa which have a clever method of generating water, and thus imply a reduced a data space. Here, ROA and NBO are combined so as to greatly reducing the dimensionality. Therefore, DQNN is tuned using the introduced RNBO for achieving better effectiveness.

Namib beetle position encoding

For attaining a finest solution in search space E, a learning parameter of DQNN Ρ is continuously tuned. It can be given by $E=\left[1\times Ρ\right]$.

Fitness function

It is a variation amid target output and DQNN output called fitness function, which can be expressed as mentioned below.

(26) $$\hbar =\frac{1}{i}\sum_{l=1}^{i}\left[T_{l}-Q_{l}\right]{\;}^{2}$$

Here, i implies overall image samples, whereas $T_{l}$ and $Q_{l}$ are target output and output achieved from DQNN, respectively.

The steps of RNBO for training DQNN are elucidated below.

Step 1: Solution initialization

Initially, each solution of a problem is considered as beetle-initialized, wherein the initialized solution is expressed as follows:

(27) $$G=\left\{G_{1},G_{2},\ldots ,G_{d},\ldots ,G_{f}\right\}$$

Here, $G_{d}$ indicates a dth candidate solution and f signifies the count of variables in the problem, whereas the population is represented by G.

Step 2: Evaluating objective function

The variation among the target output and an attained output from DQNN is termed as objective function. It is formulated using Equation (26).

Step 3: Suitability of each region for collecting water

Each problem finds a solution or else a beetle is initially positioned in the random space of the problem and estimated utilizing the objective function. Here, each of the beetles assess large values with respect to the objective function and have high capabilities for collecting water and moisture. From this viewpoint, the beetle in the question is situated in an optimal region and this optimal region can be attractive for the other beetles, which guides them to the areas for collecting water. Each area wherein a beetle such as ${Ν}_{d}$ is placed could receive various beetles. This can be computed utilizing the below equation.

(28) $$F_{d}=F_{m}\times \sin\left(\frac{\hbar \left({Ν}_{d}\right)-\hbar_{n}}{\hbar_{n}-\hbar_{m}}\times \frac{\pi }{2}\right)$$

Here, $F_{d}$ denotes the capability of beetle counts in a region, where beetle ${Ν}_{d}$ is located. $F_{m}$ is the maximal capacity of beetle counts, which can be in one region. $\hbar \left({Ν}_{d}\right)$ signifies a competence of beetle ${Ν}_{d}$, whereas $\hbar_{m}$ and $\hbar_{n}$ are the minimum as well as maximum capabilities of the beetle population, respectively. By evaluating the value of $F_{d}$, various random solutions are generated in the problem space for each of the iterations. These solutions are referred to as newer solutions as well as beetles, which search for water and are able to choose any of the real beetles, and then move towards them. Here, the $F_{d}$ values for two beetles having $\hbar_{m}$ and $\hbar_{n}$ competence is zero, as well as for $F_{m}$, respectively. The calculation of $F_{d}$ for each beetle reveals how the beetle could be observed by other beetles and how it attracts other beetles. In this association, F represents an overall beetle population that is seeking water.

(29) $$F=\sum_{d=1}^{f}F_{d}=F_{1}+F_{2}+\ldots +F_{f}$$

(30) $$F=\sum_{d=1}^{f}F_{m}\times \sin\left(\frac{\hbar \left({Ν}_{d}\right)-\hbar_{n}}{\hbar_{m}-\hbar_{n}}\times \frac{\pi }{2}\right)=F_{m}\times \sin\left(\frac{\hbar \left({Ν}_{d}\right)-\hbar_{n}}{\hbar_{m}-\hbar_{n}}\times \frac{\pi }{2}\right)+\ldots +F_{m}\times \sin\left(\frac{\hbar \left({Ν}_{f}-\hbar_{n}\right)}{\hbar_{m}-\hbar_{n}}\times \frac{\pi }{2}\right)$$

A non-linear value is incremented by merits and incremented from zero as well as $F_{m}$.

Step 4: Moving towards the wet regions

Each beetle or optimization problem resolver requires to choose regions with enough moisture for finding water. This can be considered as each of the beetles has attraction, which is similar to moisture and thus the beetle is attracted to this region. This kind of attraction to receive moisture decreases with an increase in distance. Let us consider a beetle such as ${Ν}_{d}$ in a single region and another beetle ${Ν}_{r}$ in the problem searching space. $F_{d}$ refers to the count of beetles, which aims to move towards beetle ${Ν}_{d}$. Let us initially calculate a distance among two beetles which can be represented by

(31) $$c_{dr}=\left\|{Ν}_{d}-{Ν}_{r}\right\|=\sqrt{\sum_{a=1}^{f}{\left({Ν}_{d,a}-{Ν}_{r,a}\right)}^{2}}$$

Beetle ${Ν}_{d}$ is assumed to enter a region where beetle ${Ν}_{d}$ is located.

(32) $$H\left(Μ\right)=ℑ\times H_{0}\times \exp\left(-c_{dr}^{h}\right)$$

$H_{0}$ refers to an amount of initial humidity in the problem space and atmosphere, which is equal to 2, and $H\left(Μ\right)$ denotes the moisture amount, where ${Ν}_{r}$ comes from the regions of beetle ${Ν}_{d}$. h signifies power and at this condition $c_{dr}$ implies the distance among two beetles or else the problem solution. The coefficient of increasing humidity with regard to proximity is computed and, in accordance with the increase in iteration of the developed technique, it increases as well as reaches a value wherein the coefficient value is considered as equal to 2. Due to the increasing repetition, the beetle feels a high level of moisture as it moves towards the other beetle. Hence, e increasing in iterations make a nature search go from local to global.

(33) $$ℑ=e_{m}-e_{0}\left(1-\frac{Ι}{{Ι}_{m}}\right)rand\;\left(0,1\right)$$

In this observation, Ι implies the present iteration count of the algorithm, whereas ${Ι}_{m}$ denotes the maximal iteration count of the algorithm. $e_{0}$ is an initial coefficient of increasing humidity and ℑ represents a coefficient value of increasing humidity experience by the beetles closer to the regions, which have high humidity.

The beetles experience high moisture in the final iteration owing to optimum solution proximity, or else by having high moisture, which can be absorbed by means of high coefficient in the region wherein optimum beetles have higher moisture absorption. An attraction mechanism of one beetle to another beetle wherein the present location of a single beetle and the coefficient of moisture sensing are utilized is shown below.

(34) $${Ν}_{r}^{Ε}={Ν}_{r}^{Χ}+H\left({Ν}_{d}-{Ν}_{r}^{Χ}\right)+levy$$

(35) $${Ν}_{r}^{Ε}={Ν}_{r}^{Χ}+H*{Ν}_{d}-H*{Ν}_{r}^{Χ}+levy$$

(36) $${Ν}_{r}^{Ε}={Ν}_{r}^{Χ}\left(1-H\right)+H*{Ν}_{d}+levy$$

From Remora,

(37) $$U^{N+1}=U^{N}+Β$$

where

(38) $$Β=A*\left(U^{N}-\Upsilon *U_{best}\right)$$

(39) $$A=2*J*rand\left(0,1\right)-J$$

Let

(40) $$U^{N+1}={Ν}_{r}^{Ε}$$

(41) $$U^{N}={Ν}_{r}^{Χ}$$

(42) $$U_{best}={Ν}_{r}^{best}$$

Then, Equation (37) becomes

(43) $${Ν}_{r}^{Ε}={Ν}_{r}^{Χ}+\left(2*J*rand\left(0,1\right)-J\right)*\left({Ν}_{r}^{Χ}-\Upsilon *{Ν}_{r}^{best}\right)$$

(44) $${Ν}_{r}^{Ε}={Ν}_{r}^{Χ}+2*J*rand\left(0,1\right)*{Ν}_{r}^{Χ}-2*J*rand\left(0,1\right)*J*{Ν}_{r}^{best}-J*{Ν}_{r}^{Χ}+J*\Upsilon *{Ν}_{r}^{best}$$

(45) $${Ν}_{r}^{Ε}={Ν}_{r}^{Χ}\left(1+2*J*rand\left(0,1\right)-J\right)-2*J*rand\left(0,1\right){Ν}_{r}^{best}*\Upsilon +J*\Upsilon {Ν}_{r}^{best}$$

(46) $${Ν}_{r}^{Ε}={Ν}_{r}^{Χ}\left(1+2*J*rand\left(0,1\right)-J\right)+J*\Upsilon *{Ν}_{r}^{best}\left(1-2rand\left(0,1\right)\right)$$

(47) $${Ν}_{r}^{Χ}=\frac{{Ν}_{r}^{Ε}-J*\Upsilon *{Ν}_{r}^{best}\left(1-2rand\left(0,1\right)\right)}{1+2*J*rand\left(0,1\right)-J}$$

Substituting Equation (47) by Equation (36),

(48) $${Ν}_{r}^{Ε}=\left(\frac{{Ν}_{r}^{Ε}-J*\Upsilon *{Ν}_{r}^{best}\left(1-2rand\left(0,1\right)\right)}{1+2*J*rand\left(0,1\right)-J}\right)\left(1-H\right)+H*{Ν}_{d}+levy$$

(49) $${Ν}_{r}^{Ε}-\frac{{Ν}_{r}^{Ε}\left(1-H\right)}{1+2*J*rand\left(0,1\right)-J}=\frac{\left(2rand\left(0,1\right)-1\right)J*\Upsilon *{Ν}_{r}^{best}*\left(1-H\right)+\left[H*{Ν}_{d}+levy\right]\;\left(1+2*J*rand\left(0,1\right)-J\right)}{1+2*J*rand\left(0,1\right)-J}$$

(50) $$\frac{\left(1+2*J*rand\left(0,1\right)-J-1+H\right){Ν}_{r}^{Ε}}{1+2*J*rand\left(0,1\right)-J}=\frac{\left(2rand\left(0,1\right)-1\right)J*\Upsilon *{Ν}_{r}^{best}*\left(1-H\right)+\left[H*{Ν}_{d}+levy\right]\;\left(1+2*J*rand\left(0,1\right)-J\right)}{1+2*J*rand\left(0,1\right)-J}$$

The updated equation of RHBO is represented by

(51) $${Ν}_{r}^{Ε}==\frac{\left(2rand\left(0,1\right)-1\right)J*\Upsilon *{Ν}_{r}^{best}*\left(1-H\right)+\left[H*{Ν}_{d}+levy\right]\;\left(1+2*J*rand\left(0,1\right)-J\right)}{2*J*rand\left(0,1\right)-J+H}$$

Here, Υ signifies the Remora factor.

(52) $$J=2*\frac{N}{N_{m}}$$

In Equation (51), ${Ν}_{r}^{best}$ implies the best location, $rand\;\left(0,1\right)$ represents a random number among $\left(0,1\right)$, ${Ν}_{r}^{Ε}$ and ${Ν}_{r}^{Χ}$ are the new and current positions of the beetle, respectively, whereas levy specifies the random vector, which can be calculated by

(53) $$levy=\frac{ο}{{\left|V\right|}^{\frac{1}{\varsigma }}}\left|\frac{\Gamma \left(1+\varsigma \right)\times \sin\;\left(\frac{\pi }{2}\right)\;\varsigma }{\Gamma \left(\frac{1+\varsigma }{2}\times \varsigma \times 2^{\frac{\varsigma -1}{2}}\right)}\right|$$

Here, ς is constant equal to 1.5 while ο and V are two individual random vectors, which alter in an interval $\left(0,1\right)$.

Step 5: Move the wet mass

The beetles are able to detect the regions with high moisture thanks to their smelling sense. For modelling this behaviour, the beetles’ centre of gravity and wet regions employed are utilized. The beetles search for space among gravity points and the most optimum solution. Here, it is assumed that each of the beetles has a certain quantity of water as well as moisture, which can be utilized from these waters’ centre of gravity for a more optimal search. Thus, a space offering a broader choice for detecting water could be in the regions of optimality and gravity.

(54) $${Ν}_{d}^{Ε}={Ν}_{d}^{Χ}+rand\times \left({Ν}^{*}-\bar{Ν}\right)+levy$$

${Ν}^{*}$ is a position of high moisture and $\bar{Ν}$ is a water gravity position, which is in the environment and in the beetles’ bodies. In this relationship, $\bar{Ν}$ signifies population gravity point value and, for additional influencing, the ${Ν}^{*}$ role can be increased whereas Ν can be decreased and therefore in the final iteration it is possible to perform many local searches for suitable answers.

(55) $$\bar{Ν}=\frac{\sum_{d=1}^{f}{Ν}_{d}}{f}=\frac{{Ν}_{1}+{Ν}_{2}+\ldots +{Ν}_{f}}{f}$$

(56) $${Ν}_{d}^{Ε}={Ν}_{d}^{Χ}+rand\left(e.{Ν}^{*}-\left(1-e\right)\bar{Ν}\right)+levy$$

Step 6: Hunting or removal of population

Beetles attempt to return back to their nest after watering, being positioned on a hilltop and gathering moisture from the air. During this procedure, a few are hunted by lizards. This makes it possible to provide a probability to remove any beetle on the basis of solution elimination. Generally, unsuitable solutions are eliminated from the population over time. Inappropriate solutions are highly prone to elimination and hunting; thus, random solutions are generated in the space of problems.

Step 7: Termination

The above elucidated steps are continued for attaining better solution. Algorithm 1 reveals a pseudo code of RNBO.
**Algorithm 1.** Pseudo code of RNBO.**SL. No****Pseudo code of RNBO****1**$\mathrm{Set}\;\mathrm{the}\;\mathrm{parameters}\;{Ι}_{m},H_{0}$ and so on**2**$\mathrm{Each}\;\mathrm{of}\;\mathrm{the}\;\mathrm{solutions}\;\mathrm{is}\;\mathrm{regarded}\;\mathrm{as}\;a\;\mathrm{Namib}\;\mathrm{beetle}G=\left\{G_{1},G_{2},\ldots ,G_{d},\ldots ,G_{f}\right\}$**3**Initialize the population of the Namib beetle together with random locations**4**Evaluate the fitness function using Equation (26)**5*****While*** $\left(Ι<={Ι}_{m}\right)$  ***do*****6**$\hbar_{m}=m\left(\hbar \right),\;\hbar_{n}=n\left(\hbar \right)$**7**An initial coefficient of humidity increases using Equation (33)**8**The benefits of each region for collecting water**9*****For***  d=1 to f ***do*****10**The capacity for receiving various beetles is identified utilizing Equation (28)**11*****End for*****12*****For***  d=1 to f ***do*****13*****For***  $r=F_{d}$ to f ***do*****14**$\mathrm{Searching}\;\mathrm{around}\;{Ν}_{d}$**15*****End for*****16*****End for*****17*****For***  d=1 to f ***do//***moving to wet regions**18*****For***  r=1 to f ***do*****19**$\mathrm{Compute}\;c_{dr}$$,\;H\left(Μ\right)$$\;\mathrm{and}\;{Ν}_{r}^{Ε}$ using Equations (31), (32) and (51)**20*****End for*****21*****End for*****22*****For***  d=1 to f ***do//***Calculating population mass and move towards wet mass using Equation (54)**23*****End for*****24**Ι=Ι+1**25*****End while*****26**$\mathrm{Return}\;\mathrm{finest}\;\mathrm{solution}\;\mathrm{from}\;\hbar_{n}=n\left(\hbar \right)$

### 3.6. COVID-19 Lung Infection Classification Using RNBO_DNFN

COVID-19 disease spreads as a type of pneumonia that causes air sacs inflammation in a single or both of the lungs and fills up with pus or fluid, which makes breathing challenging for the patient. Here, lung infection classification of COVID-19 is executed employing DNFN which is tuned by RNBO. An input considered for this phase is denoted by $Q_{l}$.

#### 3.6.1. Architecture of DNFN

DNFN [26] is a fusion of a deep neural network (DNN) and fuzzy logic. A deep neuro fuzzy optimizer is employed for an effectual optimization of cost as well as peak reduction. This is an integrated technique wherein a DNN is used firstly and thereafter fuzzy logic is employed in the second stage for computing system goals. An entire system consists of a few layers, namely input, hidden and output. An input layer is based on a number of input parameters along with values of fuzzification in the system. Hidden layers consist of three kinds, namely rule, normalization and defuzzification layer. An output is considered as a defuzzification layer. The crucial parameters in this system are the premises and consequents of a system. The premise parameters include time, load and pricing tariff, whereas the consequent parameters include cost as well as peak reduction. Here, premises are basics for membership functions in fuzzification at the input layer, which specifies the occurrence level in behaviours of customers or energy consumption patterns. The consequent parameters correspond to the defuzzification procedure. This task addresses two main goals, namely minimization of cost and peak reduction. The mathematical expressions for these parameters are interpreted below.

Each input or output parameters are mapped to a certain node or entity in a neuro fuzzy network of individual layers. Each input degree is assigned between 0 and 1 in accordance with the criteria stated by the fuzzy model. Each entity in the first layer is followed by the output value. Assuming two premises $x_{d}$ and $y_{d}$ as well as one consequent $z_{d}$, is expressed in the below equation.

(57) $$CD_{1,p}=\zeta XY_{p}\left(\tau_{a}\right)\;\mathrm{or}\;CD_{1,p}=\zeta UV_{p-2}\left(\tau_{b}\right)\;,\;\forall p=1,2,3,4$$

Here, $\tau_{a}$ and $\tau_{b}$ are the inputs for each pth entity, whereas ζXY and $\zeta UV_{p-2}$ represent functions of antecedent membership. $CD_{1,p}$ indicates membership degree. The functions of the membership are stated as bell shape functions, which are allotted with maximal and minimal values of 1 and 0.

(58) $$\zeta XY_{p}\left(\tau_{b}\right)=\frac{1}{1+{\left|\frac{\tau_{b}-ij_{p}}{gh_{p}}\right|}^{2\alpha_{p}}}$$

The premise parameters of membership functions that are optimized regarding training are specified by $\alpha_{p},\;\;ij_{p}$ and $gh_{p}$.

The rule base layer is the second layer, which is utilized to elucidate rule sets. All entities in this layer multiply the linguistic variable values for the satisfaction of the membership degree. A multiplication of membership variable values signifies a firing strength of the rule as interpreted in the following equation.

(59) $$CD_{2,p}=\psi_{p}=\zeta XY_{p}\left(\tau_{a}\right)\zeta UV_{p-2}\left(\tau_{b}\right),\forall p=1,2$$

The third layer is the normalization in which all entities calculate the firing strength ratio of the pth rule having the summation of every rule’s firing strength. $\psi_{p}$ represents weight, a generic network parameter. The result of all rules is thereafter normalized by means of the rules’ firing strength, which is illustrated below.

(60) $$CD_{3,p}=\bar{\psi_{p}}=\frac{\psi_{p}}{\psi_{1}+\psi_{2}},\forall p=1,2$$

The fourth layer is referred to as defuzzification layer, wherein all the rules’ consequents are calculated to present the total result on an output. This is modelled by

(61) $$CD_{4,p}=\bar{\psi_{p}}\varepsilon_{p}^{*}{}_{}=\bar{\psi_{p}}\left(lm_{p}\tau_{a}+rs_{p}\tau_{b}+wx_{p}\right),\forall p=1,2$$

Here, lm,  rs and wx are the group of consequent parameters. After that, the last layer is thought of as a summation layer, which estimates the summation of prior layer outcomes. The following equation describes the procedure of the last outcome computation.

(62) $$CD_{5,p}=\sum_{p}\bar{\psi_{p}}\varepsilon_{p}^{*}{}_{}=\frac{\sum_{p}\psi_{p}\varepsilon_{p}^{*}{}_{}}{\sum_{p}\psi_{p}}$$

**Figure 4:**
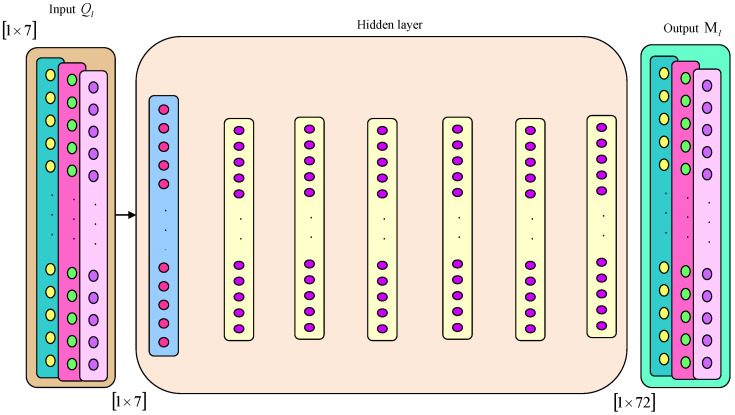
The architecture of DNFN.

An output from DNFN can be specified as ${Μ}_{l}$ and the architecture of DNFN is shown in Figure 4.

#### 3.6.2. Training of DNFN Utilizing Proposed RNBO

DNFN is presented for efficient cost and load optimization, which also addresses uncertain features of affected patients. The DNFN utilized for the second level of classification is trained employing the devised RNBO. However, RNBO is the combination of ROA and NBO. The steps followed for the proposed RNBO have already been explained in Section 3.5.2.

## 4. Results and Discussion

The outcomes of RNBO_DQNN and RNBO_DNFN are discussed in this section along with a description of the dataset, experimentation setup as well as outcomes and comparison assessment.

### 4.1. Experimentation Setup

An execution of newly framed approaches for COVID-19 lung infection identification and classification is implemented in the Python tool in a PC equipped with Windows 10 OS and 8 GB RAM.

### 4.2. Description of Dataset

The utilized dataset [19] comprised 349 CT images of positive cases and 397 of negative cases for COVID-19 belonging to 216 patients. This dataset is open source for the public to encourage CT imaging-based examination of COVID-19.

### 4.3. Experimentation Results

**Figure 5:**
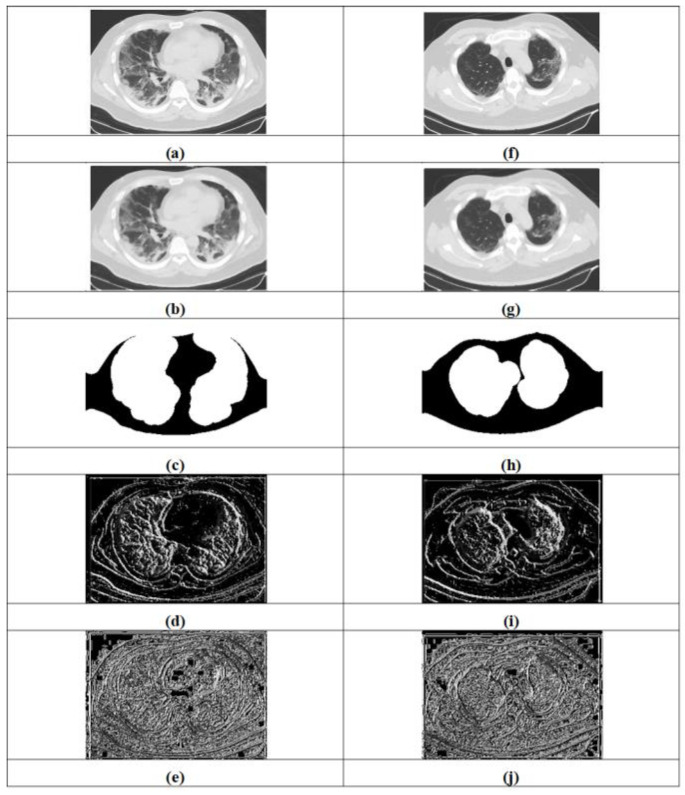
Experiment outcomes, (**a**) input image-1, (**b**) filtered image-1, (**c**) segmented image-1, (**d**) LTP-1, (**e**) LGXP-1, (**f**) input image-2, (**g**) filtered image-2, (**h**) segmented image-2, (**i**) LTP-2, (**j**) LGXP-2.

The experimentation results obtained by the proposed approaches for COVID-19 lung infection identification and classification are expounded in Figure 5. Figure 5a–e illustrate input image-1, filtered image-1, segmented image-1, LTP-1 and LGXP-1, whereas input image-2, filtered image-2, segmented image-2, LTP-2 and LGXP-2 are demonstrated in Figure 5f–j.

**Figure 6:**
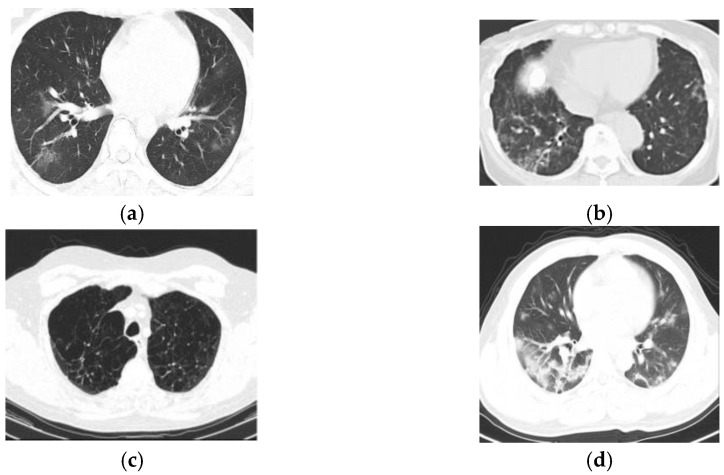
Experiment outcomes, (**a**) false negative, (**b**) false positive, (**c**) true negative, (**d**) true positive.

Figure 6 shows the experimentation results of obtained by the proposed approaches for COVID-19 lung infection identification and classification. Figure 6a denotes the false negative result, Figure 6b indicates the false positive result, the true negative result is represented in Figure 6c and the true positive result is indicated in Figure 6d.

### 4.4. Performance Measures

The newly framed techniques RNBO_DQNN and RNBO_DNFN are estimated for their performance on the basis of metrics, namely true positive rate (TPR), testing accuracy and true negative rate (TNR).

#### 4.4.1. TPR

TPR is defined as the corrected predictions of positive lung infected cases in an outcome and can be illustrated by

(63) $$P_{R}=\frac{T_{pv}}{T_{pv}+F_{nv}}$$

Here, $P_{R}$ implies TPR, $T_{pv}$ signifies true positive, whereas $F_{nv}$ symbolizes false negative.

#### 4.4.2. TNR

TNR indicates the result of the approach which precisely predicts negative lung infected classes, which can be formulated by

(64) $$N_{R}=\frac{T_{nv}}{T_{nv}+F_{pv}}$$

where $N_{R}$ specifies TNR, $T_{nv}$ denotes true negative and $F_{pv}$ implies false positive.

#### 4.4.3. Testing Accuracy

Testing accuracy can be found by the nearness of the measured value to the standard or given value and can be modelled by

(65) $$A_{R}=\frac{T_{pv}+T_{nv}}{T_{pv}+T_{nv}+F_{pv}+F_{nv}}$$

where $A_{R}$ indicates testing accuracy.

### 4.5. Comparative Techniques

The proposed techniques, namely RNBO_DQNN and RNBO_DNFN, are compared with a few existing approaches such as Inf-Net [29], U-Net [2], cascade CNN [10] and transfer learning [6] for proving the efficacy of the framed techniques.

### 4.6. Comparative Analysis

A comparison analysis is carried out amongst the devised methods and classical approaches with respect to evaluation measures for two levels of classifications.

#### 4.6.1. Assessment Based upon First Level of Classification

An assessment for the first level of classification is performed regarding performance metrics by altering the learning set and values of the k-group.

(i) Analysis based upon learning set

**Figure 7:**
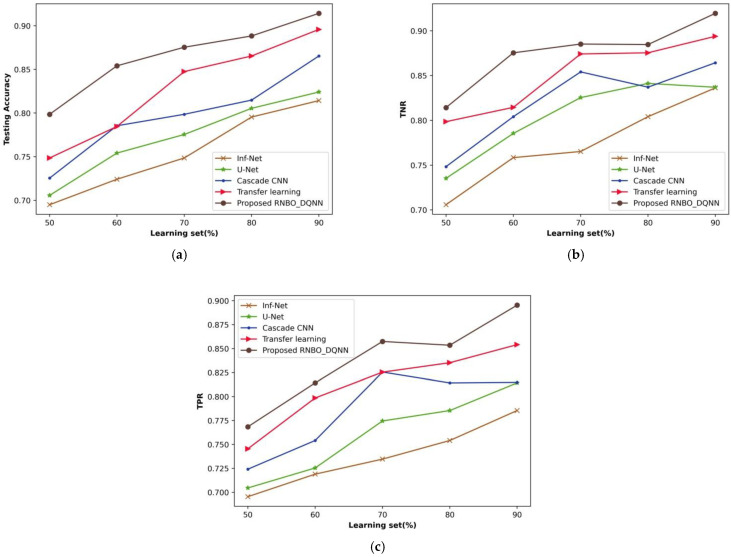
Analysis based upon learning set, (**a**) Testing accuracy, (**b**) TNR, (**c**) TPR.

A comparative evaluation of RNBO_DQNN with respect to performance metrics by changing the percentages of the learning set from 50% to 90% is demonstrated in Figure 7. An estimation of RNBO_DQNN in terms of testing accuracy is expounded in Figure 7a. A testing accuracy attained by RNBO_DQNN is 0.875 where Inf-Net, U-Net, cascade CNN and transfer learning attained 0.749, 0.775, 0.799 and 0.848, respectively, for the learning set percentage at 70%. Figure 7b shows an estimation of RNBO_DQNN with regard to TNR. When the learning set is at 70%, the TNR obtained by RNBO_DQNN is 0.885 while Inf-Net, U-Net, cascade CNN and transfer learning achieve 0.765, 0.825, 0.854 and 0.874, respectively. An evaluation of RNBO_DQNN considering TPR is elucidated in Figure 7c. The TPR achieved by RNBO_DQNN is 0.857 when Inf-Net, U-Net, cascade CNN and transfer learning obtain 0.735, 0.775, 0.826 and 0.825, respectively, with the learning set = 70%. In addition, an enhancement of performance for RNBO_DQNN based on testing accuracy with the learning set = 70% while comparing Inf-Net, U-Net, cascade CNN and transfer learning results in 14.496%, 11.423%, 8.781% and 3.187%, respectively. For a percentage of learning set = 70%, the performance enhancement of RNBO_DQNN in terms of TNR while comparing Inf-Net, U-Net, cascade CNN and transfer learning results in 13.552%, 6.755%, 2.290% and 1.250%, respectively. While the learning set = 70%, the performance improvement of RNBO_DQNN regarding TPR for Inf-Net is 14.320%, for U-Net is 9.670%, for cascade CNN is 3.722 and for transfer learning is 3.737.

(ii) Analysis based on k-group

**Figure 8:**
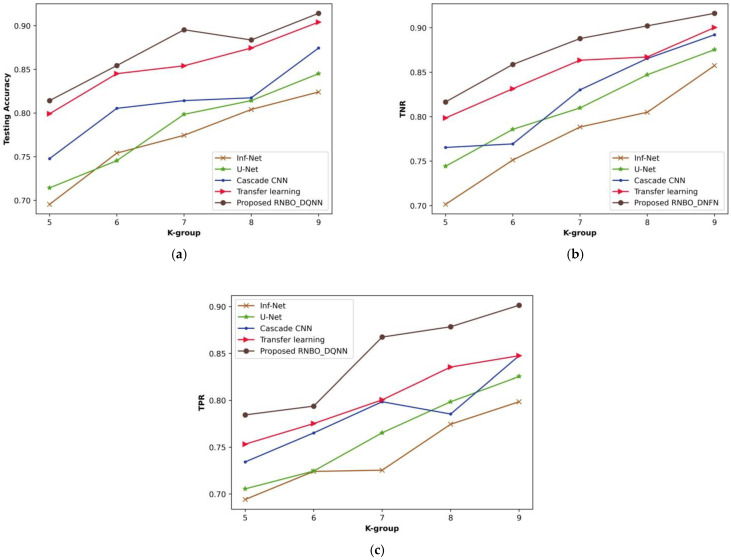
Analysis based upon k-group value, (**a**) testing accuracy, (**b**) TNR, (**c**) TPR.

Figure 8 illustrates a comparative evaluation of RNBO_DQNN regarding performance metrics by varying the k-group values from 5 to 9. An estimation of RNBO_DQNN based on testing accuracy is expounded in Figure 8a. A testing accuracy attained by RNBO_DQNN is 0.895 where Inf-Net is 0.775, U-Net is 0.799, cascade CNN is 0.814 and transfer learning is 0.854 for a value of k-group = 7. Figure 8b delineates an estimation of RNBO_DQNN by considering TNR. When the k-group value = 7, the TNR achieved by RNBO_DQNN is 0.891 while Inf-Net, U-Net, cascade CNN and transfer learning obtain 0.794, 0.827, 0.824 and 0.877, respectively. An assessment of RNBO_DQNN with respect to TPR is shown in Figure 8c. The TPR attained by RNBO_DQNN is 0.867 where Inf-Net is 0.725, U-Net is 0.765, cascade CNN is 0.799 and transfer learning is 0.801, while the value of k-group = 7. Moreover, an enhancement of performance for RNBO_DQNN regarding testing accuracy when the k-group value = 7, when comparing Inf-Net, U-Net, cascade CNN and transfer learning, results in 13.502%, 10.819%, 9.064% and 4.611%, respectively. For a k-group value = 7, the performance enhancement of RNBO_DQNN considering TNR when comparing with Inf-Net is 10.915%, with U-Net is 7.281%, with cascade CNN is 7.595% and with transfer learning is 1.567%. When the k-group value = 7, the performance enhancement of RNBO_DQNN regarding TPR when comparing Inf-Net, U-Net, cascade CNN and transfer learning results in 16.374%, 11.763%, 7.944% and 7.714%, respectively.

#### 4.6.2. Assessment Based upon Second-Level Classification

An evaluation for second-level classification is performed with respect to performance metrics by altering the learning set and values of the k-group.

(i) Analysis based on learning set

**Figure 9:**
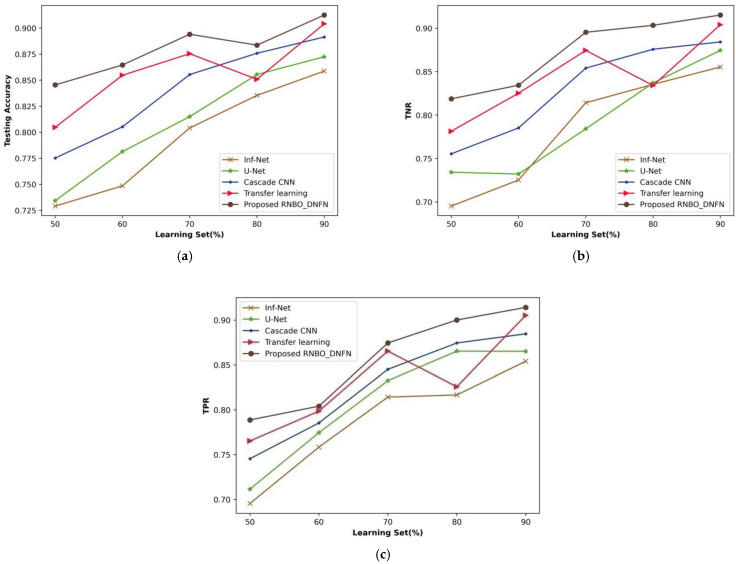
Evaluation based on learning set, (**a**) testing accuracy, (**b**) TNR, (**c**) TPR.

A comparing estimation of RNBO_DNFN regarding performance metrics by differing learning set percentages from 50% to 90% is revealed in Figure 9. An estimation of RNBO_DNFN considering testing accuracy is shown in Figure 9a. The testing accuracy achieved by RNBO_DNFN is 0.894 where Inf-Net is 0.804, U-Net is 0.815, cascade CNN is 0.855 and transfer learning is 0.875 for a percentage of learning set = 70%. Figure 9b interprets an evaluation of RNBO_DNFN considering TNR. When the learning set = 70%, the TNR obtained by RNBO_DNFN is 0.895 while Inf-Net, U-Net, cascade CNN and transfer learning obtained 0.814, 0.784, 0.854 and 0.875, respectively. An assessment of RNBO_DNFN based on TPR is described in Figure 9c. The TPR acquired by RNBO_DNFN is 0.875 while Inf-Net is 0.814, U-Net is 0.833, cascade CNN is 0.845 and transfer learning is 0.865 when the learning set = 70%. Furthermore, a performance improvement for RNBO_DNFN with respect to testing accuracy when the learning set = 70% while comparing Inf-Net, U-Net, cascade CNN and transfer learning results in 10.066%, 8.849%, 4.330% and 2.093%, respectively. For a learning set = 70%, the performance improvement of RNBO_DNFN regarding TNR while comparing with Inf-Net is 9.064%, with U-Net is 12.418%, with cascade CNN is 4.611% and with transfer learning is 2.334%. When the learning set = 70%, the performance enhancement of RNBO_DNFN based on TPR while comparing with Inf-Net is 6.891%, with U-Net is 4.799%, with cascade CNN is 3.360 and with transfer learning is 1.041.

(ii) Analysis based on k-group

**Figure 10:**
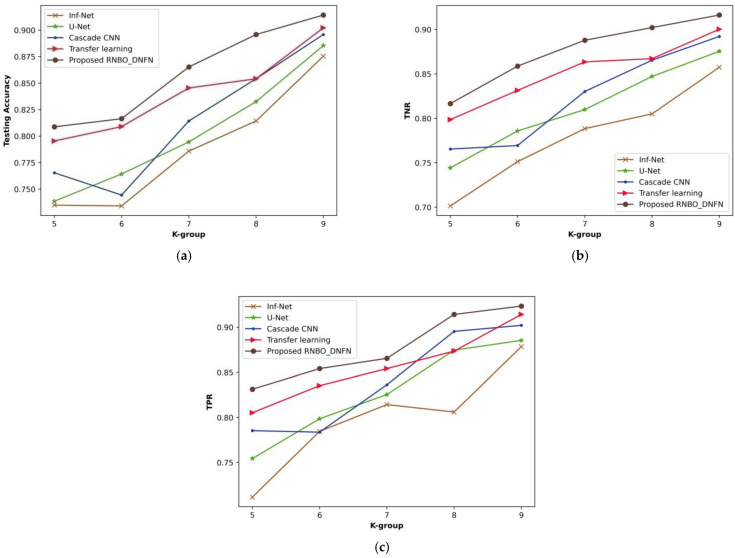
Assessment based upon k-group value, (**a**) testing accuracy, (**b**) TNR, (**c**) TPR.

Figure 10 delineates a comparative assessment of RNBO_DNFN considering performance metrics by varying k-group values from 5 to 9. The evaluation of RNBO_DNFN in terms of testing accuracy is illustrated in Figure 10a. A testing accuracy attained by RNBO_DNFN is 0.865 where Inf-Net is 0.786, U-Net is 0.795, cascade CNN is 0.814 and transfer learning is 0.845 for a value of k-group = 7. Figure 10b demonstrates an assessment of RNBO_DNFN with regard to TNR. While k-group = 7, the TNR attained by RNBO_DNFN is 0.888 while Inf-Net, U-Net, cascade CNN and transfer learning achieve 0.788, 0.810, 0.830 and 0.864, respectively. An evaluation of RNBO_DNFN regarding TPR is delineated in Figure 10c. The TPR achieved by RNBO_DNFN is 0.865 whereas Inf-Net is 0.814, U-Net is 0.825, cascade CNN is 0.836 and transfer learning is 0.854, with the value of k-group = 7. Additionally, the performance enhancement for RNBO_DNFN respective to testing accuracy while the value of k-group = 7 when comparing Inf-Net, U-Net, cascade CNN and transfer learning is 9.179%, 8.174%, 5.893% and 2.292%, respectively. For k-group = 7, the performance enhancement of RNBO_DNFN regarding TNR when comparing with Inf-Net is 11.200%, with U-Net is 8.785%, with cascade CNN is 6.492% and with transfer learning is 2.740%. While the k-group value = 7, the performance improvement of RNBO_DNFN with regard to TPR when comparing Inf-Net, U-Net, cascade CNN and transfer learning is 5.917%, 4.627%, 3.376% and 1.311%, respectively.

### 4.7. Comparative Discussion

A discussion based on the comparative analysis for RNBO_DQNN and RNBO_DNFN is shown in the tables below. Table 1 shows the values attained by comparing the assessment of RNBO_DQNN and Table 2 interprets the comparative analysis performed for RNBO_DNFN. From the below delineated tables, it is understood that RNBO_DNFN acquired maximal testing accuracy, with a TNR and TPR of 89.4%, 89.5% and 87.5% while considering the learning set = 70%. Moreover, the training time and testing time of the devised approach is 20 s and 0.2 s, respectively.

## 5. Conclusions

COVID-19, by spreading globally, has become a worldwide health crisis with numerous positive cases. Lung infection region segmentation from CT scan images has high potential in helping COVID-19 diagnosis as well as healthcare. Moreover, it is simultaneously important and challenging to select suitable classifiers to address lung feature spaces properly. Thus, COVID-19 lung infection identification and classification approaches called RNBO_DQNN and RNBO_DNFN are framed effectively. Here, CT lung images are considered and then pre-processed utilizing adaptive Wiener filtering, wherein ROI extraction is also carried out. Lung lobe segmentation is conducted on the basis of PSP-Net, which is then given for the extraction of feature phases. The features, namely LTP, LGXP, GLCM features and statistical features such as mean, kurtosis, skewness, variance and standard deviation are extracted. In the first level of classification, extracted features are classified as COVID-19 or non-COVID-19 using DQNN, which is trained by RNBO. Furthermore, RNBO is devised by the combination of ROA and NBO. If a classified output is COVID-19, then the second level of classification is performed by DNFN which is tuned by RNBO to classify further categories of affected areas as low, moderate and high infection affecting the lungs. Moreover, RNBO_DNFN achieved maximal testing accuracy, maximal TNR and maximal TPR values of 89.4%, 89.5% and 87.5%, respectively. As a future objective, the system will be extended for quantifying severe cases of pneumonia utilizing other classifiers.

## Figures and Tables

**Table 1:** Comparative discussion of RNBO_DQNN.

Analysis Based upon	Metrics/ Methods	Inf-Net	U-Net	Cascade CNN	Transfer Learning	Proposed RNBO_ DQNN
***Learning set = 70%***	*Testing Accuracy*	74.9%	77.5%	79.9%	84.8%	87.5%
	*TNR*	76.5%	82.5%	85.4%	87.4%	88.5%
	*TPR*	73.5%	77.5%	82.6%	82.5%	85.7%
***K-group = 7***	*Testing Accuracy*	77.5%	79.9%	81.4%	85.4%	89.5%
	*TNR*	79.4%	82.7%	82.4%	87.7%	89.1%
	*TPR*	72.5%	76.5%	79.9%	80.1%	86.7%

**Table 2:** Comparative discussion of RNBO_DNFN.

Analysis Based on	Metrics/ Methods	Inf-Net	U-Net	Cascade CNN	Transfer Learning	Proposed RNBO_ DNFN
***Learning set = 70%***	*Testing Accuracy*	80.4%	81.5%	85.5%	87.5%	**89.4%**
	*TNR*	81.4%	78.4%	85.4%	87.5%	**89.5%**
	*TPR*	81.4%	83.3%	84.5%	86.5%	**87.5%**
***K-group = 7***	*Testing Accuracy*	78.6%	79.5%	81.4%	84.5%	86.5%
	*TNR*	78.8%	81%	83%	86.4%	88.8%
	*TPR*	81.4%	82.5%	83.6%	85.4%	86.5%

## Data Availability

Not applicable.
